# Bioactive Coatings on Ti–Zr–Nb Alloy: Synthesis, Characterization and Implantology Potential

**DOI:** 10.3390/ma19122534

**Published:** 2026-06-11

**Authors:** Kseniia Kovalenko, Kostiantyn Sukhyi, Marcel Fedak, Miroslav Rimar, Oleh Kalinichenko, Oleksandr Yeromin, Olesia Shmychkova, Andrii Kulikov, Stanislav Kovalyov, Mykhailo Sukhyi

**Affiliations:** 1 Faculty of Manufacturing Technologies with the Seat in Presov, Technical University of Kosice, Bayerova 1, 08001 Presov, Slovakia; marcel.fedak@tuke.sk (M.F.); miroslav.rimar@tuke.sk (M.R.); andrii.kulikov@tuke.sk (A.K.); 2 Faculty of Food and Chemical Technologies, Ukrainian State University of Science and Technologies, 49000 Dnipro, Ukraine; ksukhyy@gmail.com; 3 Department of Fuel, Polymer, and Polygraphic Materials Technologies, Faculty of Nutritional and Chemical Technologies, Ukrainian State University of Science and Technologies, 49000 Dnipro, Ukraine; 4 Department of Ecology, Heat Engineering, Occupational Safety and Health, Faculty of Mechanical Engineering and Environmental Protection, Ukrainian State University of Science and Technologies, 49000 Dnipro, Ukraine; 5 Department of Physical Chemistry, Faculty of Nutritional and Chemical Technologies, Ukrainian State University of Science and Technologies, 49000 Dnipro, Ukraine; 6 Department of Power Engineering, Faculty of Computer Science and Engineering, Ukrainian State University of Science and Technologies, 49000 Dnipro, Ukraine

**Keywords:** implants, titanium, zirconium, niobium, calcium-phosphate coatings, plasma electrolytic oxidation, SBF test

## Abstract

This research reports on the properties of oxide-ceramic coatings produced by plasma electrolytic oxidation in novel electrolyte solutions for implantology applications. A series of bioactive calcium-phosphate coatings was synthesized on medical-grade Ti-13Zr-13Nb alloy using the plasma electrolytic oxidation (PEO) method. Novel electrolytes enriched with calcium and phosphorus were developed, enabling the formation of coatings with tailored physicochemical and structural characteristics. A correlation was established between the electrolyte composition and the phase composition, thickness, morphology, porosity, and microhardness of the resulting coatings. The optimum coatings exhibited a Ca/P ratio close to that of natural human bone tissue, homogeneity, a well-developed porous surface topography, and controlled resorption behavior. For the first time, a mechanism of calcium-phosphate coating resorption in a biologically active environment has been proposed. It involves partial dissolution, the formation of apatite-like surface structures, and the subsequent controlled release of Ca and P ions. In vitro testing in simulated body fluid indicated the potential bioactivity of the synthesized coatings. The proposed calcium-phosphate coatings may be considered promising candidates for future implant surface modification. The results obtained are significant for the development of advanced orthopedic and dental implants, including those fabricated using additive manufacturing technologies.

## 1. Introduction

Titanium and its alloys are among the most widely used materials in modern orthopaedic and dental implantology due to their excellent combination of mechanical strength, corrosion resistance and biocompatibility [1,2,3]. Their ability to form a stable passive oxide layer in physiological environments ensures long-term resistance to corrosion and prevents the release of harmful metal ions into surrounding tissues. In recent years, particular attention has been paid to β-type titanium alloys containing non-toxic alloying elements such as zirconium and niobium. These alloys exhibit a significantly lower elastic modulus compared to conventional α or α + β titanium alloys, which reduces the mechanical mismatch between implant and bone and minimizes the stress-shielding effect responsible for bone resorption and implant loosening [4,5].

Ti–Zr–Nb alloys are especially attractive for biomedical applications due to their favorable mechanical properties, excellent corrosion resistance and absence of potentially toxic elements such as aluminium or vanadium. Numerous studies have demonstrated that zirconium and niobium enhance both the mechanical performance and biocompatibility of titanium alloys, making them suitable candidates for long-term implantation [1,2,3,4]. Nevertheless, despite their intrinsic advantages, titanium-based implants are generally regarded as bioinert materials. While they are well tolerated by the human body, their surfaces do not actively stimulate bone tissue formation or chemical bonding with the surrounding bone matrix [6].

Osseointegration of untreated titanium implants is therefore largely governed by mechanical interlocking rather than chemical attachment. This limitation becomes particularly critical in cases of poor bone quality, high cyclic loading or long-term implantation, where insufficient bonding at the bone–implant interface may lead to micromotion, fibrous tissue formation and eventual implant failure [7]. Consequently, surface modification strategies aimed at improving the biological performance of titanium implants have become a central topic in biomaterials research.

Among various surface modification approaches, the deposition of bioactive calcium-phosphate (Ca–P) coatings has proven to be one of the most effective strategies to enhance implant osseointegration. Calcium-phosphate compounds, particularly hydroxyapatite, constitute the main inorganic component of natural bone and exhibit excellent biocompatibility and osteoconductivity. The presence of Ca–P phases on implant surfaces promotes osteoblast adhesion, proliferation and differentiation, while also facilitating direct chemical bonding between the implant and bone tissue [8,9]. As a result, Ca–P coatings are widely regarded as a key element in the development of bioactive implant surfaces.

Conventional techniques used to deposit calcium-phosphate coatings include plasma spraying, sol–gel methods, electrophoretic deposition and biomimetic precipitation. Although plasma spraying has been extensively applied in clinical practice, it is associated with several disadvantages, such as insufficient coating adhesion, non-uniform thickness, high residual stresses and partial amorphization of hydroxyapatite during high-temperature processing [10,11]. These drawbacks can lead to coating delamination or uncontrolled dissolution in physiological environments, thereby limiting long-term implant performance.

In this context, plasma electrolytic oxidation (PEO), also known as micro-arc oxidation, has emerged as a promising alternative surface modification technique for titanium and its alloys. The PEO process is based on the application of high voltages in an electrolytic environment, which leads to dielectric breakdown of the initially formed oxide layer and the generation of localized micro-plasma discharges on the surface of the substrate. These micro-discharges are characterized by extremely high local temperatures and pressures, enabling the formation of thick, ceramic-like oxide layers with strong adhesion to the underlying metal and a well-developed porous morphology [12].

For titanium-based materials, PEO coatings typically consist of titanium dioxide (TiO_2_) in anatase and rutile phases, with their relative proportions determined by process parameters such as voltage regime, current density, treatment time and electrolyte composition [13,14]. Compared to conventional anodic oxidation, PEO allows the formation of significantly thicker coatings with superior mechanical stability, wear resistance and corrosion protection, making them particularly suitable for demanding biomedical applications [9].

One of the most important advantages of the PEO technique lies in its ability to incorporate functional elements directly from the electrolyte into the growing oxide layer. When electrolytes enriched with calcium and phosphorus species are used, Ca^2+^ and PO_4_^3−^ ions are transported into the molten oxide regions generated by micro-plasma discharges and become embedded within the ceramic matrix [9,15,16,17,18,19]. This in situ incorporation mechanism enables the formation of calcium-phosphate-containing coatings without the need for additional deposition or post-treatment steps, while ensuring excellent adhesion between the coating and the metallic substrate.

The resulting Ca–P-containing PEO coatings typically exhibit a hierarchical porous structure composed of micro- and mesopores formed by discharge channels and gas evolution. Such surface morphology is highly beneficial for biomedical applications, as increased roughness and porosity enhance protein adsorption, improve cell attachment and provide favorable conditions for bone tissue ingrowth [9]. Furthermore, the porous architecture offers numerous nucleation sites for apatite formation, which is a key factor in determining surface bioactivity [9,19].

To ensure long-term clinical success, bioactive PEO coatings must meet specific structural and mechanical requirements [20]. According to current research, the optimal thickness of such coatings for orthopedic applications typically ranges from 10 to 40 μm [21,22]. This range provides an adequate barrier against ion release from the substrate while maintaining structural integrity.

Surface porosity plays a crucial role in osseointegration; an ideal porosity level of 20–50% with pore sizes ranging from 1 to 10 μm is considered optimal for enhancing protein adsorption and promoting osteoblast attachment [23,24]. In addition, the adhesive strength of the coating to the titanium substrate is a critical factor in preventing delamination during surgical implantation.

The bioactivity of Ca–P PEO coatings is most commonly evaluated using in vitro immersion tests in simulated body fluid (SBF), which closely reproduces the ionic composition of human blood plasma. The formation of apatite-like layers on the coating surface during SBF exposure is widely regarded as an indicator of the material’s ability to bond with living bone tissue in vivo. Numerous studies have demonstrated that Ca–P-containing PEO coatings can induce rapid apatite nucleation and growth, confirming their high bioactivity [19,25,26].

The mechanism of apatite formation on PEO-treated titanium surfaces is closely related to the dissolution and reprecipitation behavior of calcium-phosphate phases. Upon immersion in physiological solutions, partial dissolution of Ca–P compounds occurs, leading to the release of Ca^2+^ and PO_4_^3−^ ions into the surrounding medium. This process increases local ionic supersaturation near the implant surface, promoting heterogeneous nucleation of apatite on hydroxylated TiO_2_ surfaces. Subsequently, continuous growth of bone-like apatite layers enhances chemical bonding between the implant and bone tissue [27,28].

However, excessive or uncontrolled dissolution of calcium-phosphate phases may negatively affect coating stability and mechanical integrity. Therefore, achieving an optimum balance between bioactivity and long-term durability remains a major challenge in the development of PEO coatings. Key parameters influencing coating performance include Ca/P ratio, phase composition, coating thickness, porosity and electrolyte chemistry [29,30]. Careful optimization of these parameters is required to ensure controlled resorption behavior while maintaining sufficient mechanical strength and corrosion resistance.

In the case of Ti-Zr-Nb alloys, the presence of zirconium and niobium oxides within the PEO coating may further enhance implant performance. Zirconium oxide contributes to improved chemical stability and corrosion resistance, while niobium pentoxide is considered highly biocompatible and has been reported to positively influence osteogenic cell response and bone regeneration [29,30]. The synergistic combination of TiO_2_, Ca–P phases and alloying element oxides therefore represents a highly promising approach for the design of next-generation bioactive implant surfaces [4].

In summary, plasma electrolytic oxidation offers a versatile and effective route for the fabrication of bioactive calcium-phosphate coatings on Ti-Zr-Nb alloys. By tailoring electrolyte composition and processing parameters, it is possible to control coating structure, composition and biological performance. A comprehensive understanding of the relationships between these factors is essential for optimizing implant surface properties and ensuring long-term clinical success. In this context, the present study focuses on the synthesis and characterization of calcium-phosphate coatings on a Ti-13Zr-13Nb alloy via plasma electrolytic oxidation and evaluates their physicochemical properties, bioactivity and resorption behavior in a simulated physiological environment.

## 2. Materials and Methods

The titanium alloy Ti-13Zr-13Nb (wt.%: Nb—13.3, Zr—13.0, O—0.10, Fe—0.08, C—0.05, H—0.009, N—0.004, Ti—balance [10]) was used as the substrate material. The specimens were cylindrical, with a diameter of 25 mm and a thickness of 5 mm. Prior to coating, the samples were ultrasonically cleaned, degreased in ethanol, and rinsed with distilled water.

Oxidation was carried out in a bipolar (anodic-cathodic) pulsed current mode, with rectangular wave pulses, as this significantly stabilizes the conditions of the plasma discharge in the treatment zone [11]. The key process parameters—namely anodic/cathodic voltage (Ua, Uc), current density (j), and pulse frequency (F)—were strictly controlled, since they directly managed the energy of microdischarges and, consequently, the coating morphology. In particular, the anodic current density determines the growth rate and thickness of the layer, while the pulse frequency influences the pore size distribution and the phase transition from amorphous to crystalline structures. The introduction of cathodic pulses helped to rapidly neutralize concentration gradients arising during anodization and ensured a more uniform distribution of reaction products. This choice of electrical parameters promoted the formation of coatings with uniform thickness and composition and a reduced number of microcracks, thereby enhancing the overall quality and durability of the synthesized coatings.

Plasma electrolytic oxidation was conducted at an anodic current density of 1500 A/m^2^, cathodic current density of 750 A/m^2^ and pulse frequency 300 Hz for a duration of 5 min. The temperature of the electrolyte (2 L total volume) was kept at 10–20 °C using a water-jacketed cooling system combined with continuous magnetic stirring. The distance between the electrodes was fixed at 50 mm. After the treatment, the samples were rinsed with distilled water and air-dried. Coatings were synthesized in three series with consistent reproducibility of properties, using eight different electrolytes. The compositions and physical properties of these electrolytes are presented in Table 1.

The selection of specific electrolyte compositions in this study was driven by the need to overcome the limitations of conventional PEO electrolytes based on silicates and aluminates. Standard electrolytes provide effective ceramic-like barriers and lead to the formation of bioinert coatings with high stiffness, which can hinder rapid osseointegration. The electrolytes proposed by the authors are based on a synergistic combination of calcium and phosphorus salts, with the addition of hydroxyapatite and a complexing agent, which directly mimics the mineral component of natural bone tissue, rendering the coating presumably bioactive. The rationale for using different variations lies in the systematic modulation of the Ca/P ratio and ionic conductivity, enabling precise control of phase transitions during the microdischarge process. Unlike conventional deposition methods or standard PEO treatment, the developed electrolytes promote the formation of non-stoichiometric, presumably calcium-deficient hydroxyapatite and tricalcium phosphates. This approach provides a good bioresorption rate specifically designed for the low modulus Ti–Zr–Nb substrate, thereby creating a more favorable interface for protein adsorption and cellular signaling compared to traditional PEO coatings [16].

To ensure the statistical reliability and reproducibility of the obtained results, all measurements were carried out on a representative sample set. For each experimental series, *n* = 5 independent samples were prepared. The coating thickness and porosity were evaluated by analyzing 10 randomly selected regions on each sample using SEM micrographs and cross-sectional images. Five samples (*n* = 5) were analyzed per series, yielding a total of 50 measurement points for each series. Microhardness testing was performed with 15 indentations per sample to account for the structural heterogeneity of the ceramic-like layers. Statistical analysis was conducted using one-way analysis of variance (ANOVA), and the data are presented as mean values. This sampling protocol was adopted to achieve a 95% confidence interval, ensuring that the reported mean values accurately reflect the integral properties of the synthesized coatings.

The surface morphology and elemental composition of the samples were examined using scanning electron microscopy (SEM) with a Tescan Mira 3 LMU microscope (TESCAN, Brno, Czech Republic). To improve electrical conductivity, some samples were coated with a thin (30 nm) conductive Au layer using a Gatan 682 PECS ion sputtering system (Gatan Inc., Pleasanton, CA, USA). Images were acquired using both secondary electron (SE) and backscattered electron (BSE) detectors. The elemental composition of the coatings was determined using an Oxford Instruments X-max 80 mm^2^ spectrometer (Oxford Instruments, High Wycombe, UK).

As an alternative analysis and validation of the elemental measurement results, the mass fraction of chemical elements was also determined by non-destructive energy-dispersive X-ray fluorescence (EDXRF) using the multi-element express analyzer “Expert 02L” (Institute of Analytical Methods of Control, Kyiv, Ukraine).

The thickness of the obtained coatings was measured using an eddy current thickness gauge, FMP 10. Direct measurements of porosity were carried out with a microscope (ULAB XSP-137, (Shanghai, China) Achromatic objectives ×4 and ×10). The obtained images were processed using Image J software, version 1.46. The area of open pores was calculated and reported as a ratio to the total visible surface area.

The microhardness of the titanium alloy and the synthesized coatings was measured using a PMT-3 device (LOMO, St. Petersburg, Russia) using the Vickers indentation method with a load of 200 g. For each sample, three measurements were taken, and the average value was calculated.

The phase composition of the coatings was determined using a monochromatized X-ray diffractometer (DRON-3). A copper anode X-ray tube (Cu-Kα radiation) was used as the radiation source, and diffraction patterns were recorded in the 2θ range of 5–90°. Spectral analysis was performed using the Open Crystallography Database (COD).

In vitro testing was conducted in simulated body fluid (SBF) for 5 weeks, under thermostatic conditions at 37 ± 0.5 °C, following the procedure described in Ref. [31]. The SBF composition proposed by Kokubo was selected to ensure standardized testing conditions and to enable direct comparison with existing literature data on bioactive coatings [26]. The SBF solution was prepared by dissolving 6.057 g of tris(hydroxymethyl)aminomethane [(CH_2_OH)_3_CNH_2_] in 600 mL of distilled water. The pH was adjusted to 7.4 by adding 5 M HCl. Subsequently, the following components were added: 7.995 g NaCl, 0.353 g NaHCO_3_, 0.224 g KCl, 0.174 g K_2_HPO_4_, 0.71 g Na_2_SO_4_, 0.305 g MgCl_2_·6H_2_O, and 0.368 g CaCl_2_·2H_2_O (calculated per 1 L of final solution). Table 2 presents the ion concentrations in the simulated body fluid and human blood plasma.

The mass of the dried samples and the concentrations of Ca and P in the solutions were monitored. Calcium content was determined by titrimetric analysis with murexide, while phosphorus concentration was measured colorimetrically by the vanadomolybdophosphate method using standard analytical methods [32].

## 3. Results and Discussion

Three series of experimental samples were synthesized from each electrolyte solution, and the results listed represent averaged data. All coatings exhibited various shades of grey and displayed a uniform, rough, and porous structure. The grey appearance is attributed to the defect-rich and multiphase nature of the oxide coatings, which consist of porous non-stoichiometric TiO_2−x_ phases rather than ideal stoichiometric TiO_2_.

### 3.1. Surface Morphology and Chemical Composition

**Figure 1:**
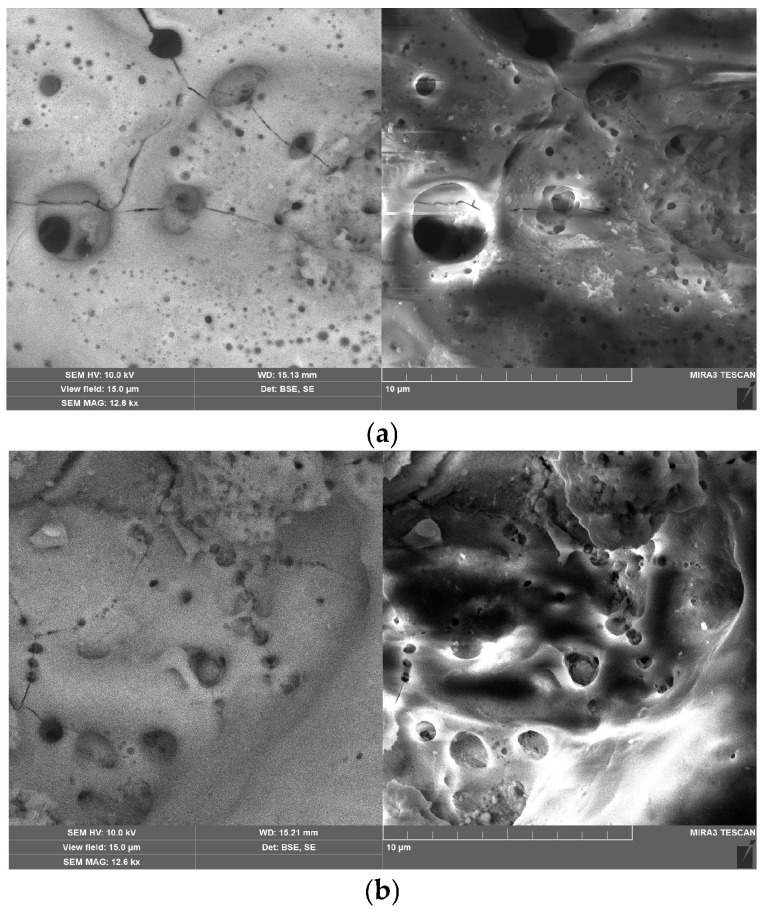

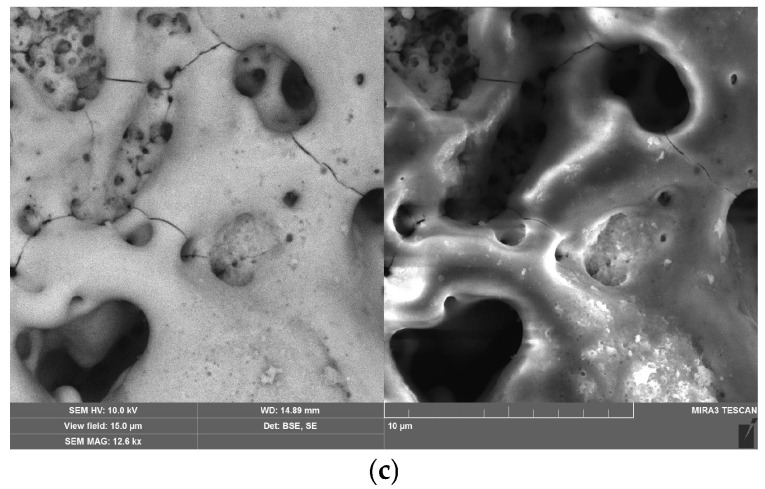
SEM micrographs of coatings obtained from electrolytes No. 3 (**a**), No. 5 (**b**), and No. 8 (**c**), showing differences in pore morphology and surface texture.

A characteristic feature of coatings produced via plasma electrolytic oxidation is the presence of surface pores of varying diameters [10]. Large pores—potential indicators of dielectric breakdown—were observed on the surfaces of samples 3 and 8 (Figure 1a,c). The surface of sample 5 exhibited a mesoporous structure (Figure 1b), which may enhance bioactivity by promoting stronger bonding with biological tissues. Cracks and structural defects were also detected in the coatings, suggesting the presence of internal stresses. These stresses are either attributed to rapid cooling following the PEO process or to abrupt temperature and voltage fluctuations during coating formation.

**Figure 2:**
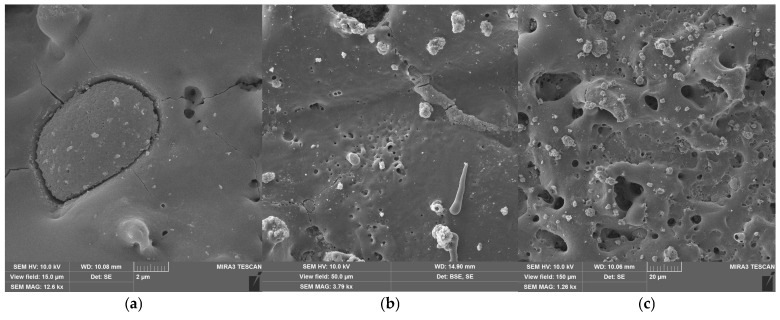
SEM images of sample 5 with a conductive gold coating at magnifications of ×15 (**a**), ×50 (**b**), and ×150 (**c**).

To enhance the secondary electron signal and obtain clearer and more contrast-rich images, sample 5 was coated with a conductive gold layer (~30 nm) prior to analysis (Figure 2).

**Figure 3:**
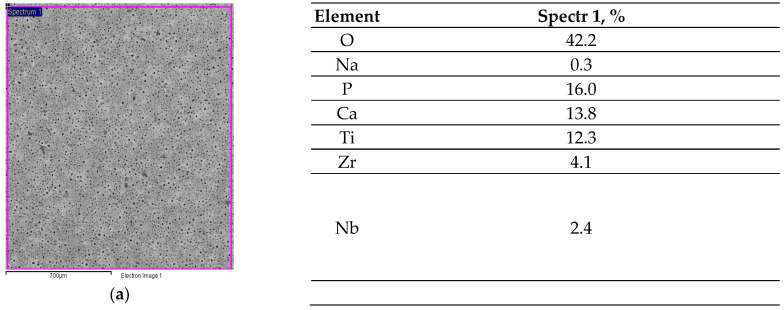

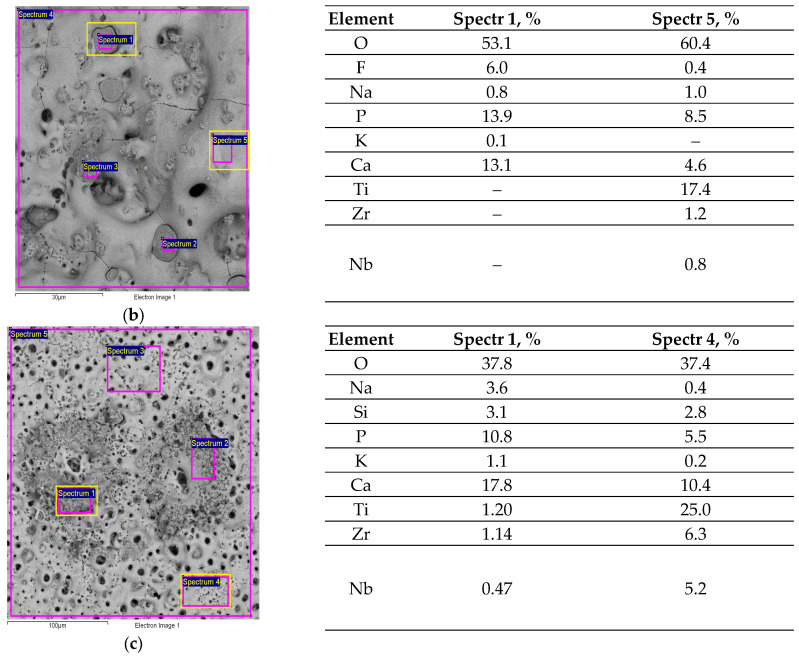
Elemental mapping based on EDS results for samples 3 (**a**), 5 (**b**), and 8 (**c**).

As shown in the figure, the surface contains inclusions of a different structure, which may correspond to calcium or phosphorus compounds. The elemental composition of the coatings was determined using X-ray microanalysis. The compositions of samples 3, 5, and 8 are presented in Figure 3.

The results showed that sample 3 has a porous and uniform structure with a homogeneous chemical composition. The Ca/P atomic ratio is 0.86, with a Ca/P atomic ratio of bone that ranges from 1.15 to 1.7, dependent on age and nutritional status of the individual. The presence of Ti, Zr, and Nb is attributed to both the metallic substrate and partially to the oxides of the coating.

Analysis of the surface composition of sample 5 confirmed the assumption that the pores were filled with calcium-phosphate compounds. A closed pore contained 13.14% Ca and 13.91% P, whereas the surrounding surface was primarily composed of metal oxides, with the calcium and phosphorus content nearly half as low. The overall Ca/P atomic ratio in the coating is 0.95, which also approaches the ratio found in human bone.

Sample 8 contains localized “nodules” that differ in composition from the surrounding coating and consist primarily of calcium and phosphorus oxides. The overall Ca/P atomic ratio is 1.96, which significantly exceeds the typical value found in natural bone tissue.

The presence of elements such as F (up to 6%), Na (up to 3.6%), Si (up to 3%), and K (up to 1%) in the composition of the titanium coatings may positively influence implant properties and enhance integration with bone tissue. For instance, fluorine is well known for its ability to stimulate bone formation [14], potentially accelerating the bonding of bone tissue to the implant. Sodium plays a key role in biological processes by regulating water–salt balance and ion exchange. Potassium is essential for cellular functions, including nerve conduction and electrolyte balance, and may enhance biocompatibility by stimulating new bone formation [15].

However, the concentrations of these elements must be carefully controlled to avoid potential risks such as toxicity, excessive hydrophilicity, or increased corrosion susceptibility. To validate the results regarding surface chemical composition, X-ray fluorescence (XRF) analysis was also performed (Table 3).

The difference in Ca/P atomic ratios measured by EDS and XRF is consistent with the different sampling depths of these techniques and with the elemental gradient typically observed in PEO layers. Due to the instrument’s limitations, elements lighter than Mg could not be detected, and thus Na and F are not listed in the table. Ti, Zr, and Nb were partially detected from the substrate. The focus was placed on Ca and P, particularly their atomic ratio. Although the ideal stoichiometric ratio for hydroxyapatite is 1.67, the values obtained for samples 3, 5, and 8 (1.34–1.60) indicate the formation of calcium-deficient hydroxyapatite (CDHA). Such non-stoichiometric calcium phosphate compounds are typical for biomimetic coatings and often exhibit enhanced bioactivity compared to pure hydroxyapatite [13]. Sample 2 had the lowest Ca/P atomic ratio at 0.36. Samples 1, 4, 6, and 7 exhibited atomic ratios around 0.6 ± 0.7, while samples 3, 8, and 5 had values closest to the target atomic ratio. These findings indicate that plasma electrolytic oxidation can be effectively used to synthesize coatings for implants with properties similar to human bone. The solutions used for synthesizing samples 5 and 8 contained hydroxyapatite, which is known to be incorporated into the coating structure [6]. Moreover, based on equilibrium constants, the formation of hydroxyapatite in solution appears more favorable than the formation of tricalcium phosphate, as shown in reactions (1) and (2) [17]:

10Ca^2+^ + 6PO_4_^3−^ + 2H_2_O → Ca_10_(PO_4_)_6_(OH)_2_ + 2H^+^, K_a_ = 6.3 · 10^116^ (1)

3Ca^2+^ + 2(PO_4_)^3−^ → Ca_3_(PO_4_)_2_, K_b_ = 2.0 · 10^28^ (2)

Titanium dioxide is formed as a result of the reaction between dispersed metallic titanium and water vapor or oxygen under high-temperature discharge conditions (reactions (3) and (4)). Its content in the coatings ranges from 35 to 63%:

Ti + O_2_ = TiO_2_ (3)

Ti + 2H_2_O_vapor_ → 800–850 °C → TiO_2_ + 2H_2_ (4)

The content of Zr and Nb oxides is 9–12 and 8–11%, respectively. These oxides are formed according to reactions (5) and (6) [16,17]:

Zr + O_2_ = ZrO_2_ (5)

4Nb + 5O_2_ = 2Nb_2_O_5_ (6)

These oxides form more slowly in alkaline electrolytes but result in more uniform and dense structures due to the absence of defects caused by local dissolution. The incorporation of Nb_2_O_5_ and ZrO_2_ oxides into the PEO coating may improve its functional properties due to the formation of a more compact and chemically inert barrier layer, which effectively suppresses the release of metal ions into the physiological environment. In particular, Nb_2_O_5_ is known for its exceptional chemical stability and high dielectric permittivity, which reduces the electronic conductivity of the oxide film, thereby contributing to long-term corrosion resistance. It also exhibits outstanding biocompatibility and has been reported to promote serum protein adsorption, facilitating the initial attachment and spreading of osteoblasts. The presence of Nb oxides has a positive regulatory effect on cell proliferation, as niobium ions released in trace amounts can stimulate osteogenic differentiation without inducing cytotoxic effects [19]. Zirconium oxide (ZrO_2_) in the coating enhances its wear resistance and stability due to a lower coefficient of friction and reduced wear rate [33]. In contrast to pure TiO_2_, the complex oxide system TiO_2_–ZrO_2_–Nb_2_O_5_ exhibits a more favorable surface energy profile, which promotes hydroxyapatite nucleation during the mineralization process. It is worth noting that the most effective calcium and phosphorus deposition was observed in alkaline solutions with pH ≥ 13.

### 3.2. Phase Analysis

**Figure 4:**
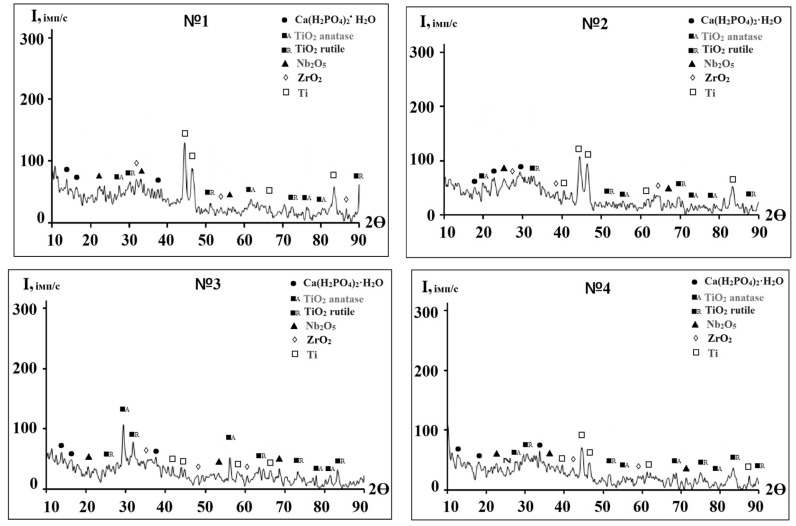

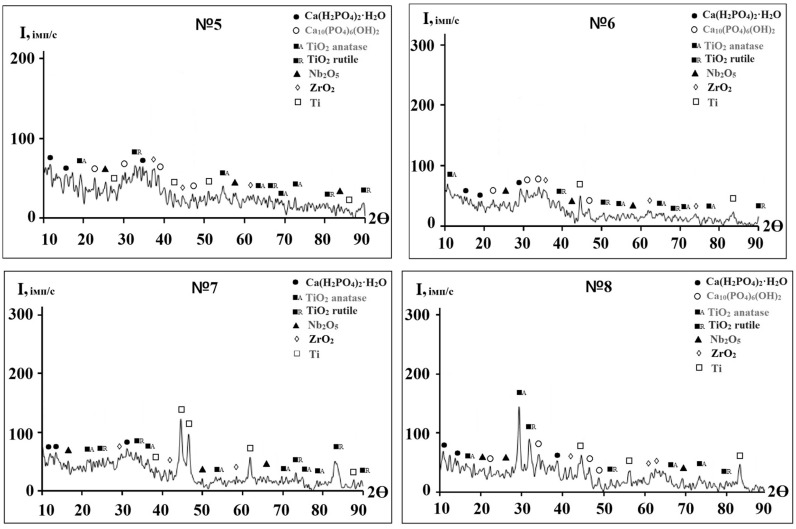
X-ray diffraction patterns of the samples.

Phase analysis of the samples was carried out using X-ray diffraction (XRD). As shown in the obtained diffractograms (Figure 4), TiO_2_ in the coatings was present in both rutile and anatase forms. Additional phases identified included ZrO_2_, Nb_2_O_5_, SiO_2_, Ca(H_2_PO_4_)_2_·H_2_O, Ca_10_(PO_4_)_6_(OH)_2_, as well as traces of metallic Ti from the substrate.

Most of the synthesized coatings were X-ray amorphous, as indicated by the presence of a broad amorphous halo in the diffractograms. Only sample 8 exhibited a mixed crystalline-amorphous phase. In these cases, the intensity of the substrate’s titanium peak decreased with increasing coating thickness, as observed in sample 6.

All coatings contained the calcium phosphate phase Ca(H_2_PO_4_)_2_·H_2_O. For samples 5, 6, and 8, distinct diffraction peaks appear on the broad amorphous background at approximately 25.8, 31.8, 32.9, 33.9, 34.1, 39.8, 46.7, and 49.5° 2θ, which are characteristic of an apatite structure, which corresponds to the international standard ICDD PDF card No. 45-0504 (as well as the reference hydroxyapatite PDF card No. 09-0432). On the surface of sample 7, hydroxyapatite was not formed, as confirmed by the absence of corresponding peaks in the diffraction pattern. Considering both the Ca/P atomic ratio and the phase composition, it can be concluded that coatings 5 and 8 are the most promising.

One of the main crystalline phases of titanium dioxide identified in the coatings—similar to conventional anodic oxidation of titanium [34]—was TiO_2_ in the anatase form, which is in good agreement with ICDD PDF cards No. 45-0504 (CDHA) and No. 21-1272 (anatase). During prolonged plasma electrolytic oxidation at elevated temperatures, crystallization may progress to a partial transformation into rutile. The high-temperature stable form of titanium dioxide, rutile, can be formed from other oxide phases (anatase and brookite) within the temperature range of 400–1000 °C [35]. During the PEO process, the high-temperature conditions within plasma microdischarges promote complex phase transformations. Along with the thermal transformation of brookite at temperatures above 600 °C, the decomposition of intermediate monetite (CaHPO_4_) phases occurs. This process leads to the formation of calcium pyrophosphate as the main product [36], which, at temperatures above 1000 °C, reacts with hydroxyapatite to form β-tricalcium phosphate, as described by reactions (7) and (8):

2HPO_4_^2−^ → P_2_O_7_^4−^ + H_2_O (7)

P_2_O_7_^4−^ + OH^−^ → 2PO_4_^3−^ + H_2_O (8)

Rutile is considered a more desirable phase compared to anatase due to its significantly lower dissolution rate in biological environments [30]. Calcium dihydrogen phosphate Ca(H_2_PO_4_)_2_·H_2_O promotes stronger adhesion between titanium and hydroxyapatite, while also reducing the solubility of the latter in acidic conditions. At temperatures above 300 °C, this compound undergoes dehydration to form γ-Ca(PO_3_)_2_, and at temperatures exceeding 450 °C, β-Ca(PO_3_)_2_ is formed. According to Hench [24], with further temperature increase, other phases may appear, including β-tricalcium phosphate or tetracalcium phosphate (Ca_4_O(PO_4_)_2_). It is noteworthy that these high-temperature, anhydrous calcium phosphate phases react with water or physiological fluids at 37 °C to form hydroxyapatite. The formation of HA on exposed surfaces of β-tricalcium phosphate occurs via the following reaction (9) [19]:

4Ca_3_(PO_4_)_2_ + 2H_2_O → Ca_10_(PO_4_)_6_(OH)_2_ + 2Ca^2+^ + 2HPO_4_^2^ (9)

As shown in Figure 4, for samples 5, 6, and 8, specific diffraction peaks appeared on a broad amorphous halo at 25.8, 31.8, 32.9, 33.9, 34.1, 39.8, 46.7, and 49.5° (2θ). Although these reflections are characteristic of the apatite structure, their significant broadening indicates a low degree of crystallinity. Considering the obtained Ca/P atomic ratios and the dissolution–precipitation mechanism described by reaction (9), it can be concluded that the synthesized phase is calcium-deficient hydroxyapatite (CDHA) or a carbonate-substituted non-stoichiometric apatite. The presence of Ca(H_2_PO_4_) ·2H_2_O and its potential dehydration products further contributes to the structural complexity of the bioactive layer, potentially enhancing implant integration with bone tissue.

### 3.3. Mechanical Properties

**Figure 5:**
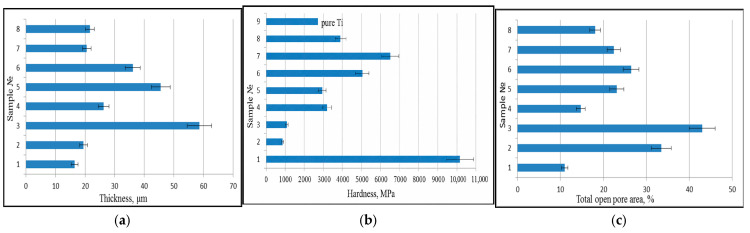
Diagrams illustrating the geometrical and mechanical properties of the samples: (**a**) thickness, (**b**) hardness, (**c**) ratio of pore area to total visible surface area.

The mechanical properties of the synthesized coatings are presented in the diagrams shown in Figure 5. The evaluated parameters included coating thickness, hardness, and the ratio of pore area to the total surface area. The data are presented as mean ± standard deviation (*n* = 5).

The highest coating thickness—50 ± 10 μm—was observed in samples 3, 5, and 6, while the remaining samples had a thickness of 20 ± 5 μm. As shown in the diagram in Figure 5b, the apparent composite microhardness of the samples varied significantly. Modern biomaterials with a hardness greater than that of bone can hinder the transfer of necessary mechanical stress to adjacent bone tissue, leading to resorption around the implant and thus weakening the structure. This biomechanical mismatch, which results in the death of bone cells, is known as the “stress shielding effect.” To ensure proper mechanical compatibility, metallic materials must also exhibit reversible deformation similar to that of biological tissues [27]. Therefore, to prevent implant loosening and extend service life—avoiding the need for revision surgery—implant materials must combine high strength with a suitable elastic modulus and reversible deformation values close to those of bone. It should also be noted that bone strength is anisotropic, i.e., it depends on the direction of the applied load [37]. Samples 2 and 3 exhibited hardness values that were 70% and 60% lower, respectively, compared to commercially pure titanium [38]. Although the elastic modulus was not directly measured, the observed decrease in microhardness, combined with the highly porous surface morphology, indirectly indicates a reduction in the effective Young’s modulus of the PEO-coated system, potentially mitigating the stress-shielding effect. This reduction is considered optimal from a biomechanical perspective, as it helps to minimize the stress shielding effect by reducing the stiffness mismatch between the metallic implant and the natural bone tissue. Such mechanical modulation promotes a more favorable stress distribution at the interface, potentially improving the long-term integrity of the bone–implant system. To reduce biomechanical mismatch and excessive hardness, the development of a porous structure—achieved mechanically or electrochemically (via etching)—is recommended. This would make implant properties more similar to bone and enhance biointegration.

The pore area-to-total surface area ratio ranged from 11 to 43%. Sample 1, the hardest, exhibited the lowest porosity, while sample 3, the thickest, had the highest. The average pore surface area was approximately 25%. Cortical bone porosity typically ranges from 5 to 15%, while trabecular bone ranges from 40 to 95% [39]. The presence of open pores has a positive impact on bone growth by allowing cells to grow inward, improving implant-bone anchorage. An increase in processing time during PEO leads to a higher coating density and a reduction in the number of open pores [16], which may negatively affect bioactivity by limiting the surface area available for apatite nucleation. Therefore, it is important to find a balance between sufficient coating thickness and the required porosity.

Based on these findings, it can be concluded that an optimum result requires a balance between adequate thickness, high porosity, and moderate hardness—achievable through appropriate process parameter selection. In this regard, sample 3 is the most favorable.

### 3.4. SBF-Test

**Figure 6:**
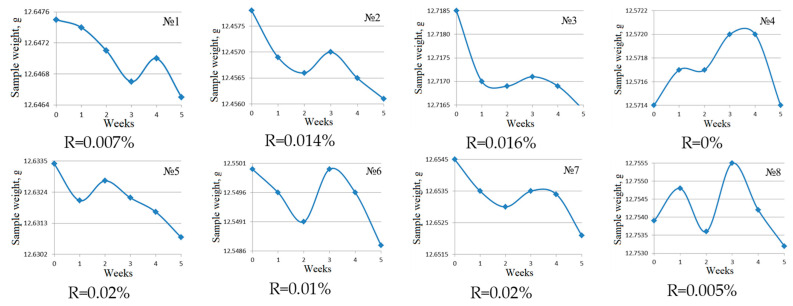
Results of the SBF test over a 5-week period indicate the percentage of resorption (R).

All obtained coatings underwent in vitro testing in simulated body fluid (SBF) to evaluate the resorption behavior and mineralization potential of the oxide layers. The results are presented in Figure 6.

**Figure 7:**
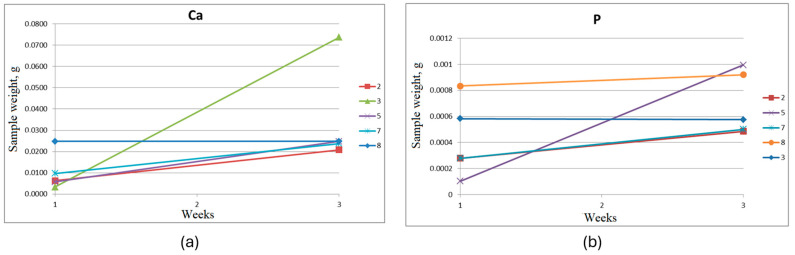
Changes in the concentration of Ca—(**a**) and P—(**b**) in the solution during the first three weeks after the SBF test, where 2, 3, and 5–8 correspond to the numbers of the tested samples.

The highest resorption rates were observed in samples 5 and 7, both showing a 0.02% mass loss. In contrast, sample 4 began gaining mass from the first week. In combination with the X-ray diffraction and electron diffraction results discussed above, this trend indicates the nucleation and gradual growth of calcium-deficient apatite structures on the surface. For samples 1–3 and 5–7, a common trend was observed: during the first two weeks, gradual dissolution of the coating occurred with the release of Ca and P ions into the solution (into the trauma zone, Figure 7).

As shown, during the active dissolution phase (weeks 1–3), the concentrations of Ca and P in the solution increased, except for calcium in sample 8 and phosphorus in sample 3. Subsequently, in weeks 2–3, a slight increase in sample mass was observed, which can be attributed to the formation of calcium-phosphate compounds on the coating surface in the physiological solution, as well as partial saturation of the pores. From weeks 3–4 until the end of the testing period, the sample mass continued to decrease. In sample 8, this process was observed to repeat twice over the five-week period, with a total weight loss of only 0.005% and a stable calcium concentration in the solution. It should be noted that increasing the sample surface area would likely result in higher Ca and P concentrations in the solution, assuming the resorption mechanism remains consistent. Although the mass fluctuates due to the periodic predominance of either dissolution or precipitation, the increasing concentrations of Ca and P in the solution (Figure 7) indicate a stable and predictable ion release rate. This cyclic process, characterized by an overall resorption rate of less than 1%, ensures long-term structural integrity of the coatings, with sample 8 exhibiting the most favorable controlled-release behavior.

Based on these findings, the resorption mechanism of calcium-phosphate coatings appears to involve the following stages: (i) partial dissolution of the coating with the release of Ca and P ions into the physiological environment; (ii) saturation of the coating with the physiological solution and subsequent formation of a calcium-phosphate layer on the surface; (iii) further controlled dissolution of the coating.

This process is likely cyclical. However, the observed coating thickness and the low overall resorption level (up to 1%) suggest that such behavior can be sustained over an extended period.

A key outcome of this study is the exceptional structural stability of the synthesized PEO coatings in a physiological environment. The observed resorption coefficient was below 1%, which is significantly lower than that of conventional bioactive surfaces. For example, hydroxyapatite coatings produced by plasma spraying often exhibit much higher dissolution rates and are prone to delamination due to the presence of metastable phases and poor interfacial bonding [40]. In contrast, the PEO process on the Ti-Zr-Nb alloy forms a graded transition zone, where the oxide layer grows directly from the substrate, ensuring excellent adhesion, which should be confirmed by testing. The low resorption coefficient, combined with the gradual formation of presumably calcium-deficient apatite layers, suggests that these coatings can provide long-term mechanical integrity, avoiding the common drawbacks of rapid degradation associated with conventional thermal spraying techniques.

## 4. Conclusions

A series of bioactive oxide-ceramic coatings was successfully synthesized on a Ti-13Zr-13Nb alloy via plasma electrolytic oxidation (PEO) using novel electrolyte compositions. The developed coatings exhibit a complex, multi-phase architecture primarily consisting of matrix oxides TiO_2_ in anatase and rutile modifications, ZrO_2_, and Nb_2_O_5_ integrated with calcium-phosphate phases, specifically Ca(H_2_PO_4_)_2_·H_2_O and presumably non-stoichiometric calcium-deficient hydroxyapatite (CDHA). The electrolyte composition exerted a decisive influence on the structural and functional properties of the layers, allowing for a targeted, application-driven selection of the optimum parameters.

Coatings synthesized from electrolyte No. 3 exhibited the most favorable biomechanical profile, characterized by a maximum thickness of 50 ± 10 μm and the highest surface porosity, 43%. This structural architecture resulted in a significant reduction in composite microhardness, making Sample No. 3 highly promising for load-bearing orthopedic implants where mitigating the stiffness mismatch (stress-shielding effect) is a primary clinical priority.

Coatings produced from electrolytes No. 5 and 8 demonstrated superior chemical bioactivity and predictable mineralization behavior. These samples achieved the optimum Ca/P atomic ratios of 1.34–1.6, closely mirroring the mineral matrix of natural human bone. Furthermore, in vitro testing in simulated body fluid (SBF) confirmed a stable, cyclic three-stage resorption mechanism with an overall mass loss of less than 1% over a 5-week period, ensuring long-term structural integrity alongside steady ion release. Consequently, instead of a single universal coating, this study proposes a comprehensive, criteria-based approach to surface design. Depending on the clinical site, Sample No. 3 is recommended for high-load applications requiring porous osteoanchorage, while Samples No. 5 and 8 possess exceptional osteoconductive potential for implant designs requiring accelerated chemical osseointegration. However, to fully validate the clinical safety and biological efficacy of these tailored surfaces, subsequent stages of this research will focus on cell proliferation, viability assays, and comprehensive in vivo evaluations.

## Figures and Tables

**Table 1:** Composition and properties of the electrolytes.

No.	Composition	Total Concentration, g/L	pH	Electrical Conductivity, μS/cm
1	Ca(H_2_PO_4_)_2_	17	6	6.4
	Ca(CH_3_COO)_2_·xH_2_O			
2	Ca(H_2_PO_4_)_2_	35	13	9.5
	NaOH			
3	Na_6_P_6_O_18_	23	6	9
	Ca(CH_3_COO)_2_·xH_2_O			
4	Ca(H_2_PO_2_)_2_	17	5	2.9
	Ca(H_2_PO_4_)_2_			
5	Na_6_P_6_O_18_	22	13	4.5
	Ca_10_(PO_4_)_6_(OH)_2_			
	Ca(H_2_PO_2_)_2_			
	KOH			
	NaF			
6	Ca_10_(PO_4_)_6_(OH)_2_	10	13	18.5
	H_3_PO_4_			
	KOH			
	ethylenediaminetetraacetic acid (EDTA)			
7	Ca_10_(PO_4_)_6_(OH)_2_	40	4	4.4
	H_3_PO_4_ (conc.)			
8	Na_6_P_6_O_18_	39	13	4.6
	Ca(CH_3_COO)_2_·xH_2_O			
	Ca_10_(PO_4_)_6_(OH)_2_			
	KOH			
	ethylenediaminetetraacetic acid (EDTA)			
	Na_2_SiO_3_			

**Table 2:** Ion concentrations in biological fluids [26].

Chemical Compound	Concentration (10^−3^ M):	
	SBF	Blood Plasma
Na^+^	142.0	142.0
K^+^	5.0	5.0
Mg^2+^	1.5	1.5
Ca^2+^	2.5	2.5
Cl^−^	147.8	103.0
HCO_3_^−^	4.2	27.0
HPO_4_^2−^	1.0	1.0
SO_4_^2−^	0.5	0.5
pH	7.40	7.2–7.4

**Table 3:** Relative elemental content on the surface of the samples (atomic ratio).

No.	Ca/P	O, %	Ti, %	Ca, %	P, %	Zr, %	Nb, %	Si, %	K, %
1	0.634	40.34	31.51	5.77	9.10	6.59	5.73	–	–
2	0.36	40.46	33.90	3.12	8.54	7.00	6.08	–	–
3	1.34	39.07	26.66	11.42	8.54	7.04	6.14	–	–
4	0.67	39.47	33.70	4.70	7.00	7.84	6.88	–	–
5	1.60	39.07	21.23	16.10	10.06	6.94	6.16	–	–
6	0.65	38.94	29.86	5.31	8.16	7.05	6.17	–	4
7	0.60	40.57	31.02	5.69	9.43	6.83	5.98	–	–
8	1.36	37.92	37.81	4.17	3.06	8.65	7.66	0.47	0.15

## Data Availability

The original contributions presented in this study are included in the article. Further inquiries can be directed to the corresponding author.

## Abbreviations

BSE	Backscattered Electron
COD	Crystallography Open Database
EDTA	Ethylenediaminetetraacetic Acid
EDS	Energy-Dispersive Spectroscopy
EDXRF	Energy-Dispersive X-ray Fluorescence
PEO	Plasma Electrolytic Oxidation
SBF	Simulated Body Fluid
SE	Secondary Electron
TLA	Three-Letter Acronym
XRD	X-ray Diffraction
